# Cerebral hemorrhagic infarction was diagnosed subsequently after high-amplitude slow waves detected on processed electroencephalogram during sedation: a case report

**DOI:** 10.1186/s40981-021-00483-3

**Published:** 2021-10-21

**Authors:** Keisuke Mihara, Haruna Nakahara, Kouhei Iwashita, Kenji Shigematsu, Ken Yamaura, Kozaburo Akiyoshi

**Affiliations:** 1 Department of Anesthesiology, Fukuoka University School of Medicine, 7-45-1 Nanakuma, Jonan-ku, Fukuoka, 814-0180 Japan; 2 Department of Anesthesiology, Fukuoka University Chikushi Hospital, 1-1-1 Zokumyouin, Chikushino, 818-8502 Japan; 3 Department of Anesthesiology and Critical Care Medicine, Kyushu University Graduate School of Medicine, 3-1-1 Maidashi, Higashi-ku, Fukuoka, 812-8582 Japan

**Keywords:** Processed electroencephalogram, Cerebral hemorrhagic infarction, High-amplitude slow waves

## Abstract

**Background:**

Continuous electroencephalogram (EEG) monitoring is useful for assessing the level of sedation and detecting non-convulsive epileptic seizures and cerebral ischemia in the intensive care unit. This report describes a case of cerebral hemorrhagic infarction diagnosed after the detection of high-amplitude slow waves on processed EEG during sedation.

**Case presentation:**

A 68-year-old man who underwent cardiac surgery was sedated in the intensive care unit following an invasive procedure. High-amplitude slow waves appeared on processed EEG monitoring before the detection of anisocoria. Computed tomography revealed a cerebral hemorrhagic infarction.

**Conclusions:**

In the management of critically ill patients, continuous EEG monitoring with forehead electrodes may be useful in the early detection of brain lesions.

## Background

Processed electroencephalogram (EEG) monitoring has been used and recommended for the assessment of the depth of anesthesia during surgery [1, 2]. Additionally, it may be used for the assessment of sedation in the intensive care unit (ICU) [3]. Multiple EEG electrodes are required to detect non-convulsive epileptic seizures and stroke, whereas their application to the forehead is required for the evaluation of sedation in processed EEG monitoring. Processed EEG monitoring is used to assess the depth of sedation in cases managed with artificial ventilation in our hospital. Furthermore, we expect to detect continuous non-convulsive epileptic seizures or cerebral ischemia, especially in critically ill patients [4]. Here, we report the case of a patient with postoperative cerebral hemorrhagic infarction, where slow waves were detected during processed EEG monitoring before the appearance of pupillary dilatation. We believe that the use of processed EEG monitoring in ICU may be useful not only for the evaluation of sedation but also for the early detection of stroke during sedation.

## Case presentation

A 68-year-old man underwent mitral valve replacement and tricuspid annuloplasty three years ago. Several weeks ago, he had shortness of breath on exertion, and was diagnosed with worsening of the residual mitral and tricuspid regurgitation, thus necessitating mitral valve replacement and tricuspid annuloplasty. His comorbidities included chronic heart failure, hyperthyroidism, and preoperative medications included losartan potassium, spironolactone, warfarin, levothyroxine sodium, and bisoprolol fumarate.

The surgery was performed as scheduled. Inotropic drugs were required to wean off the artificial cardiopulmonary device because of the low cardiac output. After surgery, the patient was admitted to the ICU and placed on artificial ventilation, and he continued to require catecholamines administration. The patient was sedated with propofol and dexmedetomidine, and the sedation was assessed by monitoring the EEG with four electrodes placed bilaterally on the forehead and processed by Sedline® (Masimo Corporation, CA, USA).

**Fig. 1:**
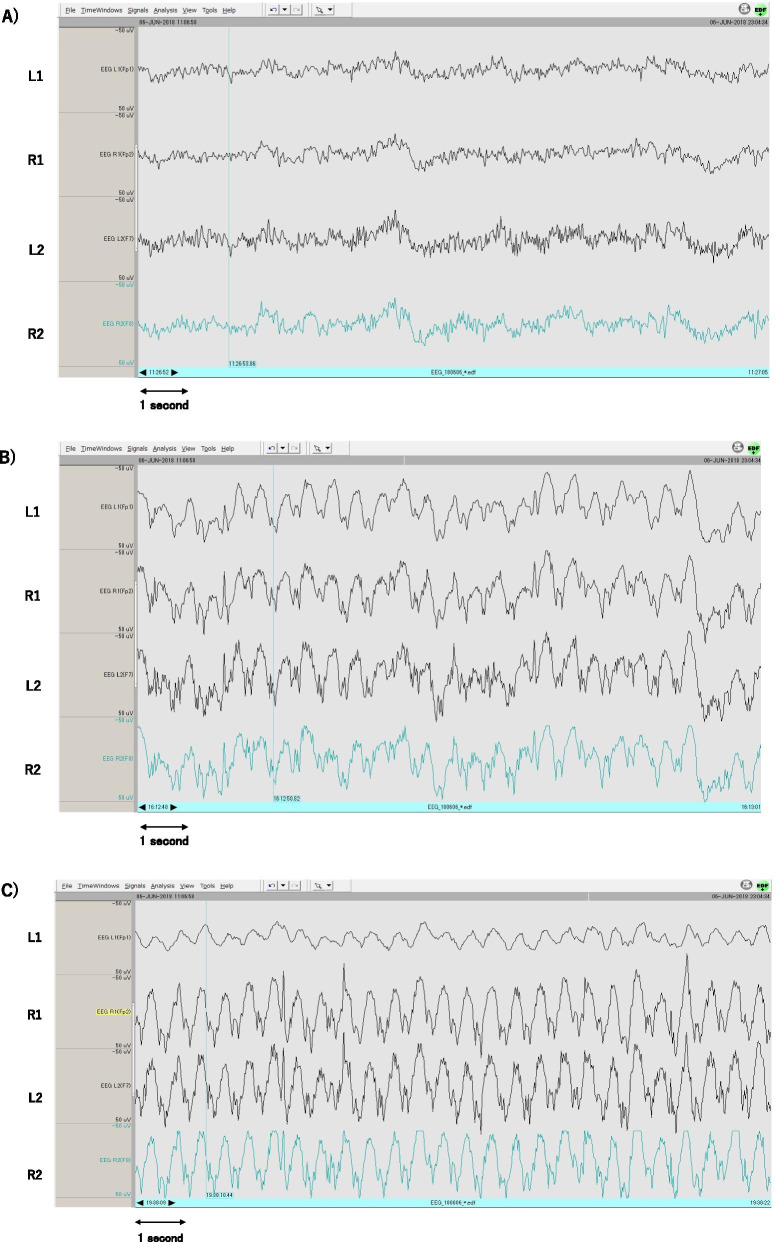
The electroencephalogram on postoperative day 7. An alpha-dominant pattern at 11 a.m. **A** changed into a delta-dominant pattern with high-amplitude on bilateral forehead at 4 p.m. **B**. Reduced amplitude with asymmetric suppressed/delta-dominant pattern detected on left forehead at 7 p.m. **C**. L1, R1, L2, and R2 are labels of the electrodes. L1 and R1 are affixed to the bilateral forehead above the eyebrows, and L2 and R2 are affixed to the hairless area at the upper region of the bilateral temples. The amplitude range is − 50 μV to 50 μV

On postoperative day (POD) 2, an intra-aortic balloon pump (IABP) was inserted to address the circulatory insufficiency, which developed despite the increased catecholamine doses. The patient also had an intrathoracic hematoma, which was removed by a re-open thoracic surgery, and continuous hemodiafiltration dialysis was initiated on the same day owing to acute renal failure. On POD 4, propofol was temporarily discontinued to attempt a spontaneous awakening trial. However, the patient’s consciousness level was still very low. He opened his eyes slightly and had an abnormal flexion, both as a response to pain stimulus at the same time. The Glasgow Coma Scale (GCS) score was 6: eye opening (E) was 2, verbal response (V) was “Tube” (T), and best motor response (M) was 3. The patient state index (PSI) value ranged between 25 and 40, and his pupils were isocoric. We did not perform the wake-up test every day because the patient was in a severe circulatory state, and the blood pressure could have become unstable if synchronization with the ventilator was disrupted by reducing propofol. On POD 6, circulatory failure due to right heart failure was determined, and veno-arterial extracorporeal membrane oxygenation (VA-ECMO) was introduced. On POD 7, the pupils remained isocoric (4.0 mm), and the light reflex was prompt. Anisocoria (left: 6 mm, right: 2 mm) was detected at 5 a.m. on POD 8, and the pupils were bilaterally dilated to 6 mm at 10 a.m. on the same day. His GCS score was 3 (E1, VT, M1), and raw EEG on SedLine® revealed slow waves, while the PSI values ranged between 20 and 25 at that time. The dose of sedative medication did not change during this period. Head computed tomography revealed an extensive left cerebral hemorrhagic infarction, with an almost flat EEG in multiple electrodes positioned bilaterally over the frontal, temporal, parietal, and occipital regions. The possibility of an embolus due to IABP or VA-ECMO was considered to be the cause of the infarction. Neurosurgeons concluded that there was no indication for surgery. We reviewed the SedLine® data with Polyman, a free software for EEG-viewer application. The raw EEG on POD 7 revealed the following significant changes: an alpha-dominant pattern at 11 a.m. (Fig. 1A); high-amplitude slow waves and a delta-dominant pattern on bilateral forehead while under sedation with propofol 2.5 mg/kg/h and dexmedetomidine 0.4 μg/kg/h at 4 p.m. (Fig. 1B); and subsequently, at 7 p.m. the amplitude of waveform on the left forehead decreased with an asymmetric suppressed/delta-dominant pattern (Fig. 1C) before the development of anisocoria. An explanation was given to the family, and the VA-ECMO was discontinued owing to a flat line EEG presentation. The patient was confirmed to have died on POD 8.

## Discussion

Herein, we report a case of high-amplitude slow delta waves, being detected during processed EEG monitoring, which was observed before the appearance of pupil dilatation in a patient with cerebral hemorrhagic infarction. The dose of sedative medication was not altered while we observed these EEG changes, and we consider it to have detected the onset of stroke.

Indications for continuous EEG monitoring include the detection of non-convulsive epileptic seizures, treatment evaluation of epileptic seizures, detection of cerebral ischemia, evaluation of sedation and therapeutic coma, and prognostic evaluation of brain damage [4]. EEG is useful for the diagnosis of acute ischemic stroke in the emergency ward [5]. Intraoperative arousal, postoperative delirium, and postoperative cognitive impairment may be reduced when the depth of general anesthesia is managed using processed EEG monitoring [6]. The SedLine® used in our case performed EEG processing using two electrodes on each side of the forehead. It showed a PSI value that indicated the depth of anesthesia, the PSI value from 25 to 50 indicates an appropriate depth of anesthesia. It can also monitor real-time raw EEGs.

EEG monitoring can detect an extensive ischemic stroke and estimate the size of the infarct lesion within hours of onset of a cerebral infarction [5]. EEG electrodes are generally placed according to a mapping system that relates the anatomy of the head surface to the underlying cortical regions of the brain. The standard EEG mapping is referred to as the 10–20 international system. This system measures the nasal root and occipital tuberosity, as well as the anterior point of the bilateral auricles, and obtains the vertex from each midpoint. The area between the nasal root and occipital tuberosity, and between the bilateral preauricular points is divided into 10% or 20%, and a total of 21 electrodes are placed systematically [7]. Standard critical care continuous EEG requires a minimum of 16 electrodes placed according to the 10–20 international system. If fewer than 16 electrodes are used, the interpretation of the critical care continuous EEG may be limited due to the inadequate spatial sampling and inability to distinguish artifacts from cerebral activity [4]. A few reports have shown that 2–7 electrodes are not sensitive enough to detect non-convulsive epileptic seizures [4, 8]. In our case, even with only four electrodes placed on the forehead, we could detect that the EEG changed to a delta wave, which is thought to appear at the onset of cerebral infarction. In cerebral ischemia, high-frequency activities, such as beta waves, are usually diminished; meanwhile, delta waves are increased on the affected side [9, 10]. Furthermore, the wave amplitudes can be increased or decreased [9]. In the present case, delta waves observed on POD 7, at 7 p.m., were initially presumed that there was a damage in the left frontal lobe or a large part of the left cerebral hemisphere because the amplitude of the waveform on the left forehead decreased. Processed EEG monitoring may allow for earlier detection of intracranial abnormalities, although it is not a sufficient substitute for subjective sedation scales such as the Richmond Agitation-Sedation Scale [11]. It was considered important for the processed EEG monitoring to possess the ability to display and record the raw EEG waveforms in addition to seeing the PSI value of sedation.

On POD 4, it was difficult to differentiate whether the patient's disturbance of consciousness was because of the confusion caused by his general condition or a small stroke. We considered the risk of moving the patient to the imaging suite to be relatively high owing to his general condition, hence, we did not perform imaging tests. At that time, EEG using the 10–20 international system should have been performed to detect abnormalities in a wide range of brain regions.

It would be difficult to detect occipital and temporal lesions, as Sedline® is a forehead monitor. Unfortunately, the fact that it only represents the lesions affecting the frontal lobe may be a limitation in detecting abnormal EEG signals. There are no reports on the sensitivity of EEG of the forehead in detecting stroke in our knowledge. However, EEG of the forehead may be useful during sedation of patients at risk of stroke, because at least when the EEG changes, immediate imaging tests can be performed to determine the indication for surgery, catheterization, or drug treatment.

## Conclusions

We experienced a case of a patient with cerebral hemorrhagic infarction, in which high-amplitude slow waves on the processed EEG monitoring appear before confirming pupillary discrepancy. Monitoring raw EEG over time, even with only electrodes in the forehead, may be useful for the early detection of stroke.

## Data Availability

Not applicable.

## Abbreviations

EEG: Electroencephalogram
ICU: Intensive care unit
POD: Postoperative day
IABP: Intra-aortic balloon pumping
GCS: Glasgow Coma Scale
PSI: Patient state index
VA-ECMO: Veno-arterial extracorporeal membrane oxygenation
